# Suture material for flexor tendon repair: 3–0 V-Loc versus 3–0 Stratafix in a biomechanical comparison *ex vivo*

**DOI:** 10.1186/s13018-014-0072-9

**Published:** 2014-08-21

**Authors:** Martin C Jordan, Stefanie Hölscher-Doht, Michael G Jakubietz, Rafael G Jakubietz, Rainer H Meffert, Karsten Schmidt

**Affiliations:** 1 Department of Trauma, Hand, Plastic and Reconstructive Surgery, Würzburg University Hospital, Oberdürrbacher Straße 6, Würzburg 97080, Germany

**Keywords:** Barbed suture, Barbed suture material, Flexor tendon repair, Knotless tendon repair, Stratafix, V-Loc

## Abstract

**Background:**

Barbed suture material offers the possibility of knotless flexor tendon repair, as suggested in an increasing number of biomechanical studies. There are currently two different absorbable barbed suture products available, V-Loc™ and Stratafix™, and both have not been compared to each other with regard to flexor tendon repair. The purpose of this study was to evaluate both suture materials for primary stability under static and cyclic loading in a biomechanical *ex vivo* model.

**Methods:**

Forty fresh porcine flexor digitorum profundus tendons were randomized in two groups. A four-strand modified Kessler suture technique was used to repair the tendon either with a 3–0 V-Loc™ or 3–0 Stratafix™ without a knot. Parameters of interest were mode of failure, 2-mm gap formation force, displacement, stiffness and maximum load under static and cyclic testing.

**Results:**

The maximum load was 42.3 ± 7.2 for the Stratafix™ group and 50.7 ± 8.8 N for the V-Loc™ group. Thus, the ultimate tensile strength was significantly higher for V-Loc™ (*p* < 0.05). The 2-mm gap occurred at 24.8 ± 2.04 N in the Stratafix™ group in comparison to 26.5 ± 2.12 N in the V-Loc™ group (n.s.). Displacement was 2.65 ± 0.56 mm in the V-Loc™ group and 2.71 ± 0.59 mm in the Stratafix™ group (n.s.). Stiffness was 4.24 ± 0.68 (N/mm) in the V-Loc™ group and 3.85 ± 0.55 (N/mm) the Stratafix™ group (n.s.). Those measured differences were not significant.

**Conclusion:**

V-Loc™ demonstrates a higher maximum load in tendon reconstruction. The differences in 2-mm gap formation force, displacement and stiffness were not significant. Hereby, the V-Loc™ has an advantage when used as unidirectional barbed suture for knotless flexor tendon repair.

## Background

The ideal flexor tendon repair has been subjected to constant change and development over the last decades. For instance, multistrand repair technique as well as the use of innovative suture material could improve the tensile strength and enable postoperative mobilization [1],[2]. To date, there is no final consensus about the ideal suture material in flexor tendon repair but it has been demonstrated that the suture material should have high tensile strength, prevent gapping, should be easy to use and be biocompatible [3],[4]. In 2009, experimental studies started to assess a new generation of commercially available barbed suture materials in tendon repair [5],[6]. These suture materials offer possible flexor tendon repair with reduction of bulk at the repair site and without redundant knot as potential weak point [5],[7]. Trocchia et al. and Parikh et al. demonstrated knotless reconstruction of flexor tendons with barbed sutures in comparison to materials like FiberWire™, Prolene™ or Ethibond™ [5],[6]. Subsequent biomechanical tests confirmed this data and evaluated various suture techniques. The suture technique for barbed material needs to ensure high suture-tissue interaction as multiple barbs have to lock inside the tendon [7]–[14], but no information is available, which type of barbed suture can provide this requirement best. All of the known studies used either V-Loc™ (Covidien, Dublin, Ireland) or Stratafix™ (Ethicon, Johnson & Johnson, USA - formerly known as Quill™ SRS, Angiotech Puerto Rico Inc.) as barbed suture but there is no direct comparison between both materials in all published data. Macroscopic, Stratafix™ appears to be more flexible and the V-Loc™ is more rigid, but it is not clear whether this makes a difference. The purpose of this study was to verify potential differences between both barbed sutures through a biomechanical *ex vivo* test.

## Methods

### Specimen

**Figure 1:**
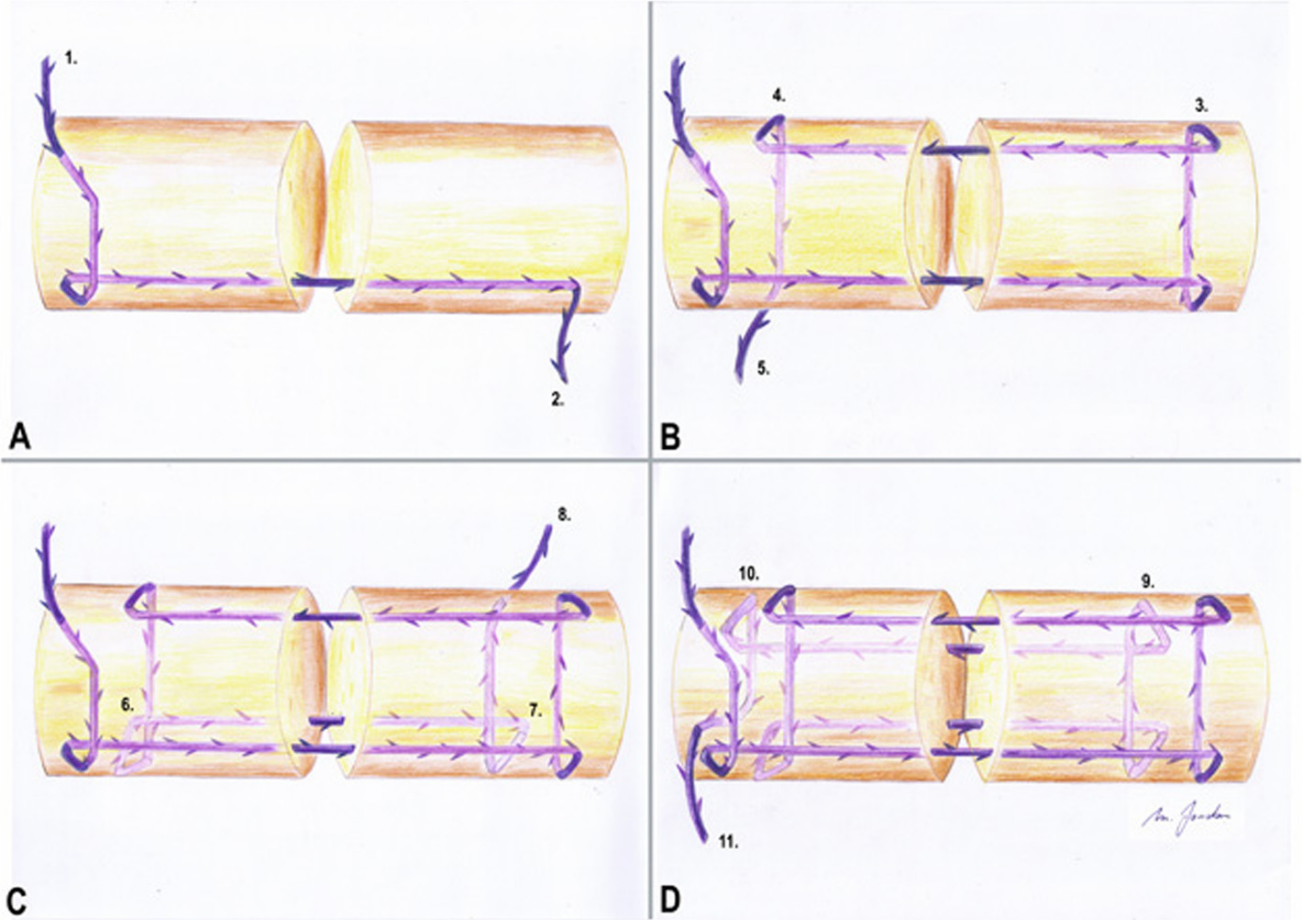
**Successive steps of knotless flexor tendon repair with a four-strand modified Kessler suture technique (A–D).** Using this technique, barbs are located in contrary direction to the tension force and are buried inside the tendon.

**Figure 2:**
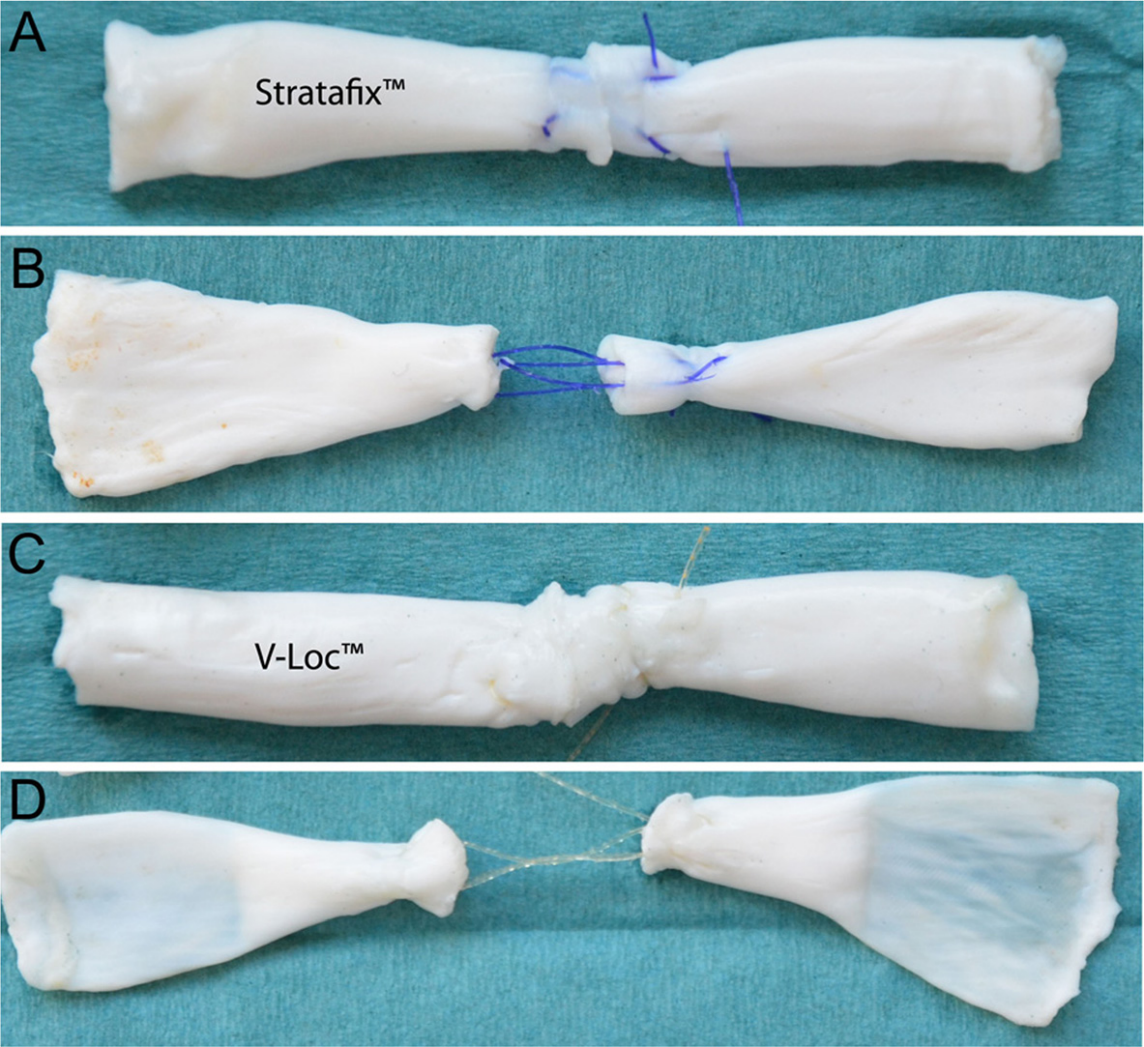
**Tendon repairs. (A, B)** Tendon repaired with Stratafix™ before and after cyclic testing. **(C, D**) Tendons repaired with V-Loc™ before and after testing.

Forty fresh frozen porcine flexor digitorum profundus tendons were used for this study. It is known that porcine flexor tendons have similar biomechanical properties to human flexor tendons, and they are frequently used in biomechanical studies [15]. They can easily be obtained in sufficient number and consistent quality. Tendons with a deviating diameter, defects like deformity, synovitis or obvious trauma, were excluded. Harvested tendons were stored inside saline-soaked swabs and deep-frozen at −20°C. Tendons were thawed at room temperature for 12 h and randomly assigned to one of two different groups (*n* = 20), V-Loc™ or Stratafix™ (Table 1). Prior to testing, the diameter and length of all flexor tendons had been measured to get equal samples. A calliper was used to measure the diameter at the repair site before and after tendon repair. A size 15 scalpel was used to carefully set the defect in the middle of each tendon. All tendons were repaired with a four-strand modified Kessler (Kirchmayr-Pennington) suture [16] with locking technique and a core suture purchase of 0.7 mm has been used (Figures 1 and 2).

**Table 1:** Groups and materials

**Group**	**Technique**	**Number**	**Testing**	**Parameters of interest**
1. Stratafix™	Kessler	10	Static	Maximum load, 2-mm gap, stiffness
2. Stratafix™	Kessler	10	Cyclic	Displacement
3. V-Loc™	Kessler	10	Static	Maximum load, 2-mm gap, stiffness
4. V-Loc™	Kessler	10	Cyclic	Displacement

Four different groups of flexor tendons were repaired with V-Loc™ or Stratafix™. Testing was static or cyclic, and parameters of interest were 2-mm gap formation force, stiffness, displacement and maximum load.

### Suture material

**Figure 3:**
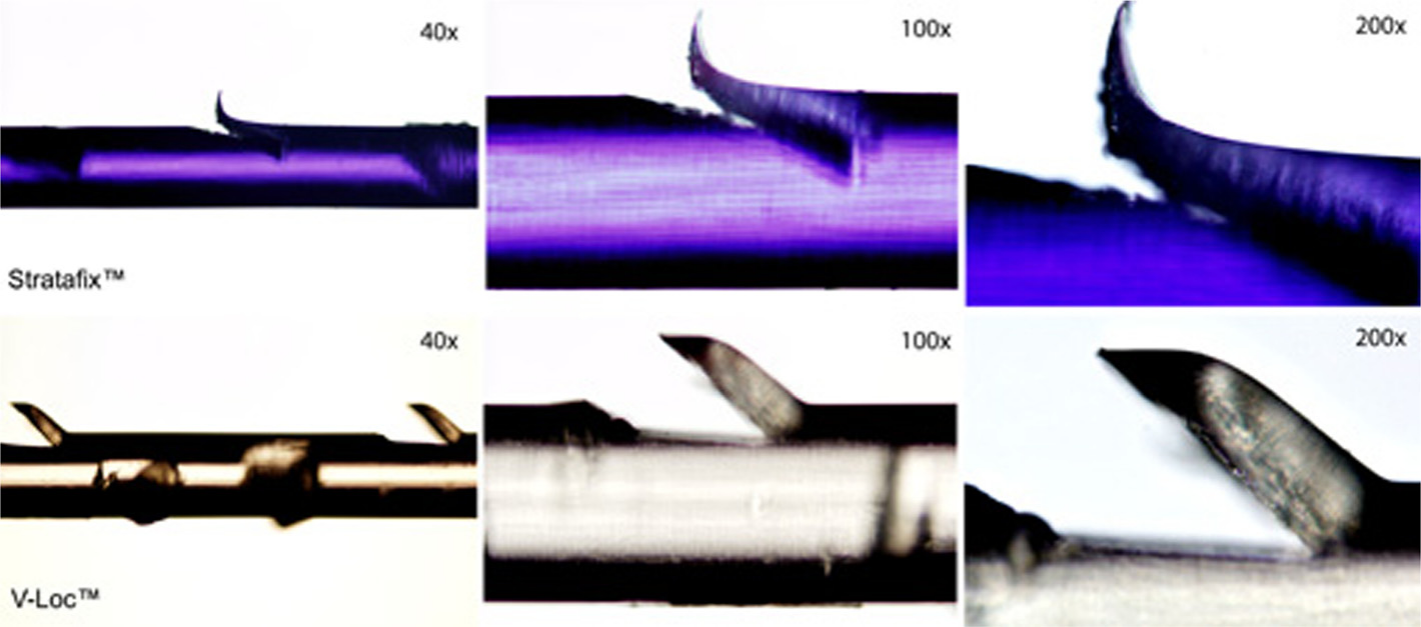
**Stratafix™ and V-Loc™ with increasing magnification.** Stratafix™ has more spiky barbs whereas V-Loc™ barbs appear more blunt. The barbs are located circumferential on each thread.

The V-Loc™ 180 (Covidien, Dublin, Ireland) is synthesized from a copolymer of glycolic acid and trimethylene carbonate and presents as an absorbable thread with circumferential barbs on its surface. We used a 3–0 U.S.P. V-Loc™ with a four-strand modified Kessler suture for tendon repair [17]. Stratafix™ Spiral PDO Device (Angiotech Puerto Rico Inc., Ethicon, formerly known as Quill™ SRS) consists of Polydiaxone (C_4_H_6_O_3_) × and is a synthetic absorbable suture where barbs can also be found circumferential around its surface. A 3–0 U.S.P. Stratafix™ has been used in this study. Stratafix™ is currently only available as bidirectional suture, so it had to be cut in between to obtain a unidirectional barbed suture. The shape of the barbs of V-Loc™ and Stratafix™ is different as seen under magnification (Figure 3) with an Olympus®-Microscope (Shinjuku-ku, Japan; Type BX 51 TF No. Oc22834).

### Biomechanical test setup

Biomechanical tests have been conducted with a mechanical testing machine (Z020, Zwick/Roell, Ulm, Germany) and the testXpert II software (Version 3.0, Zwick/Roell). Uniaxial testing was performed using a 100 N load cell and two stainless steel clamps. Pre-testing was performed to proof sufficient fixation of the tendon ends without slipping. The testing gauge length (distance between two clamps) was standardized 3 cm. Three Newton for preload was used during static and cyclic testing.

The static test was a load to failure test with an advancement rate of 20 mm/min where the 2-mm gap formation force, stiffness and the maximum load were recorded. The 2-mm gap formation force represents the tension that produces a 2-mm gap at the repair site by linear distraction and was evaluated as clinical failure. Stiffness represents the rigidity of the suture material and was measured in the load–displacement curve. The maximum load represents the ultimate tensile strength before finale failure. For finale failure, we distinguished between suture rupture and suture pullout.

The cyclic loading started with a setting stage for 15 cycles. Hereby, load between 5 and 15 N was applied. Thereafter, cyclic loading started with 250 cycles between 5 and 20 N. Load, displacement and time were continuously recorded to generate a load–displacement curve for each tendon. Out of this load–displacement curve, the displacement during the 250 cycles was measured in mm. Further, the mode of finale failure was measured. The advancement rate for cyclic testing was also 20 mm/min. The load of 20 N covers passive protected rehabilitation, and in our pre-tests, we could see 20 N as threshold before the gap formation occurred in this specific setting. A total of 250 cycles was chosen since other cyclic tendon repair studies showed 200 cycles to be sufficient [11],[18]. Our own pre-tests with 500 and 2,000 cycles confirmed these results. The displacement is a reliable parameter for comparison of different suture materials. To avoid dehydration during the cyclic loading, a saline spray was used constantly.

### Parameters of interest and statistics

The parameters of interest were type of failure (pullout vs. rupture), 2-mm gap formation force (N), displacement (mm), stiffness (N/mm) and maximum load (N) during static or cyclic testing (Table 1). All data was recorded with Excel™ (Microsoft, Redmond, WA, USA) and analysed with SPSS™ (SPSS Inc., Chicago, IL, USA). Shapiro-Wilk test and independent sample *t* test were used for comparison of the groups. A *p* value less than 0.05 was considered statistically significant. A power assessment using a significance level of 5% and a power of 80% indicated a sample size of at least *N* = 7.

## Results

### Final failure

The most common type of failure was suture rupture, seen in 39 of 40 samples. Only one suture pullout occurred in the Stratafix™ group.

### 2-mm gap formation force

**Figure 4:**
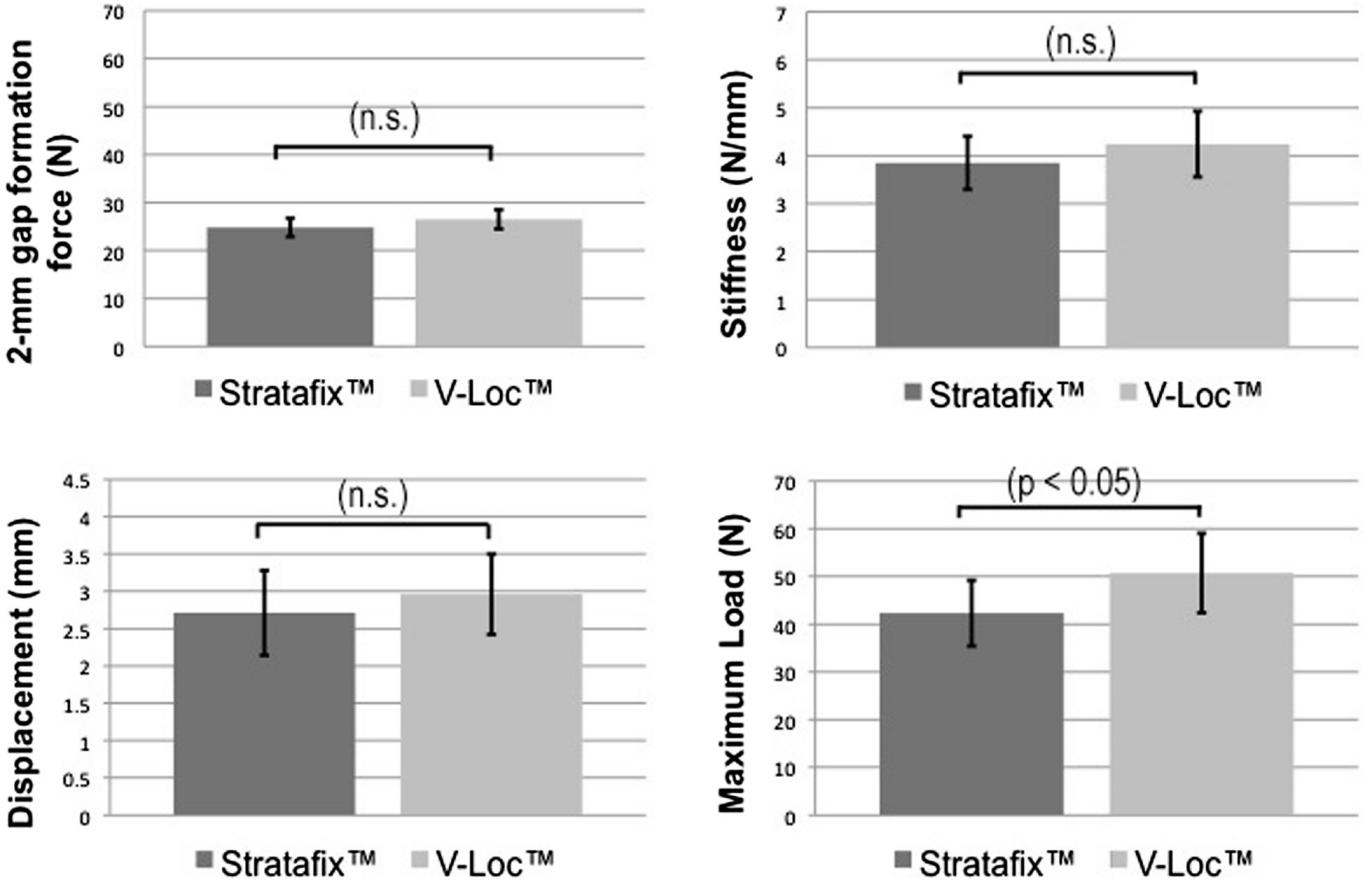
**Results of 2-mm gap formation force, displacement, stiffness and maximum load of both materials.** There is a significant difference between the maximum loads (*n.s.* not significant).

The mean force to produce a 2-mm gap was similar in both groups. Gap formation occurred at 26.5 ± 2.12 N in the V-Loc™ group and 24.8 ± 2.04 N in the Stratafix™ group. The difference in gap formation was statistically not significant (Figure 4).

### Displacement

The results were 2.65 ± 0.56 mm in the V-Loc™ group and 2.71 ± 0.59 mm displacement in the Stratafix™ group (Figure 4). The difference was not significant.

### Stiffness

Stiffness was 4.24 ± 0.68 (N/mm) in the V-Loc™ group and 3.85 ± 0.55 (N/mm) in the Stratafix™ group. The measured difference was not significant (Figure 4).

### Maximum load

The maximum load was 50.7 ± 8.8 N for the V-Loc™ group compared with 42.3 ± 7.2 N for the Stratafix™ group. The V-Loc™ can withstand significantly higher maximum load before the final failure *p* < 0.05 (Figure 4).

## Discussion

Our biomechanical *ex vivo* study compares two barbed suture materials for their use in flexor tendon repair. The maximum load occurs before suture failure, and we could show a higher ultimate tensile strength for V-Loc™ in comparison to Stratafix™. The 2-mm gap formation force represents the required load to produce a defect at the repair site and is interpreted as clinical failure. The 2-mm gap formation forces were similar in both groups as well as the stiffness. Displacement represents the increased length after cyclic loading, and the results were similar for V-Loc™ and Stratafix™.

In summary, these findings demonstrate a higher tensile strength for flexor tendons repaired with V-Loc™ instead of Stratafix™. The reason for that distinct biomechanical behaviour might be the diverse shape of the barbs of Stratafix™ and V-Loc™. Both suture materials are supposed to have similar core diameters, but the shape of the Stratafix™ barbs might be the reason for early failure. They produce a deeper cut into the thread in comparison to the V-Loc™ barbs (Figure 3). Despite this result, clinical failure can already occur at lower forces in terms of a significant gap at the repair site. Therefore, gap formation can be seen as the more important parameter. Nevertheless, if working with a knotless technique, the strongest suture should be preferred.

Knotless and non-obstructive repair for flexor tendon are reasons why barbed suture materials have been suggested by several authors [10],[11],[19],[20]. Still, clinical usage has not been established due to the lack of *in vivo* studies. The available data rely on biomechanical *ex vivo* tests that were able to demonstrate similar repair strength of barbed suture in comparison to the traditional knotted repair [7],[10],[14]. Yet, in the available data, there is no analysis about the specific suture materials. The following barbed suture materials have been used in experimental flexor tendon repair:

Troccia et al. published their research data in 2009, where they used a 2–0 barbed bidirectional non-absorbable polypropylene suture (Quill™) for flexor tendon repair. They could show similar gap formation force in comparison to the control group using Ethibond™ as suture material. The maximum load was significantly lower for the knotless repair [6]. Parkih et al. took the effort to compare a 2–0 barbed bidirectional non-absorbable polypropylene suture (Quill™) with a 4–0 Prolene™, 4–0 Ethibond™ and 4–0 Fiberwire™. A six-strand knotless suture technique using the barbed material demonstrated increased tensile strength in this study [5]. In 2011, our group authored by Zeplin et al. used a 3–0 unidirectional absorbable V-Loc™ and demonstrated comparable maximum load as a knotted tendon repair with 3–0 PDS (Ethicon™) [14]. The same year, Marreo-Amadeo et al. used a 2–0 Quill™ Polydioxanone suture and compared it to a 3–0 Surgilion™ (Davis and Geck, Norwalk, CT, USA) with no significant difference between both repair techniques [7]. McCellan et al. tested a barbed non-absorbable size 0 polypropylene (Quill™) suture and could exhibit advantages against a 3–0 PDS (Ethicon™) [10]. Peltz et al. presented a detailed study where they used a 3–0 absorbable V-Loc™. They compared it to a 3–0 silicone-coated braided polyester suture (Ticron, Syneture, Norwalk, CT, USA) and reached superior results with their knotless repair technique [5]. Afterwards, Zeplin et al. compared a 3–0 PDS™ to a 3–0 absorbable unidirectional barbed V-Loc™ and reported about the benefit of an additional 5–0 peripheral running suture (Vicryl™, Ethicon). The peripheral repair increased the maximum load of a knotless barbed suture by 63% under static and 91% under cyclic testing [13]. In 2013, Joyce et al. contributed with another study where they compared a 2–0 non-absorbable barbed V-Loc™ to a 3–0 PDS™ by using a locked cruciate technique with analogous result as other authors [9]. Comparing these studies is difficult because of different study designs and varying suture techniques. No information about the appropriate barbed suture material can be drawn out of these publications.

## Conclusion

This study proves V-Loc™ and Stratafix™ to be potentially useful in flexor tendon repair and points out a higher maximum load for V-Loc™, which should be considered when using unidirectional barbed sutures in flexor tendon repair.

In summary with the current literature, our work suggests the possibility of knotless flexor tendon repair. Nevertheless, using only a core suture without a peripheral suture probably has limitations in early active mobilization. Furthermore, *in vivo* studies are required to prove feasibility.

## Competing interests

The authors declare that they have no competing interests.

## Authors’ contributions

MCJ carried out the biomechanical testing and drafted the manuscript. SHD participated in the test design and in pre-testing. MGJ and RGJ assisted with the manuscript and test setting. RHM supervised the biomechanical testing and revised the article with helpful recommendation about illustration and statistical analysis. KS initiated the study, participated in its design and provided the material. He was also involved in previous studies and gave advise about the suture technique. All authors have read and approved the final manuscript.
